# Spatial optimization of invasive species control informed by management practices

**DOI:** 10.1002/eap.2261

**Published:** 2021-01-21

**Authors:** Makoto Nishimoto, Tadashi Miyashita, Hiroyuki Yokomizo, Hiroyuki Matsuda, Takeshi Imazu, Hiroo Takahashi, Masami Hasegawa, Keita Fukasawa

**Affiliations:** ^1^ Graduate School of Agricultural and Life Sciences University of Tokyo 1‐1‐1 Yayoi, Bunkyo‐ku Tokyo 113‐8657 Japan; ^2^ National Institute for Environmental Studies Center for Health and Environmental Risk Research 16‐2 Onogawa Tsukuba Ibaraki 305‐8506 Japan; ^3^ Faculty of Environment and Information Sciences Yokohama National University 79‐7 Tokiwadai, Hodogaya‐ku Yokohama 240‐8501 Japan; ^4^ Environmental and Community Affairs Department Nature Conservation Division Chiba Biodiversity Center Chiba Prefectural Government 955‐2 Aoba‐cho, Chuo‐ku Chiba City Chiba 260‐8682 Japan; ^5^ Japan Wildlife Research Center 3‐3‐7 Kotobashi, Sumida‐ku Tokyo 130‐8606 Japan; ^6^ Faculty of Science Toho University 2‐2‐1 Miyama Funabashi Chiba 274‐8510 Japan; ^7^ National Institute for Environmental Studies Center for Environmental Biology and Ecosystem Studies 16‐2 Onogawa Tsukuba Ibaraki 305‐8506 Japan

**Keywords:** adaptive management, decision‐making, invasive species, optimization, resource allocation, snapping turtles, state‐space model, uncertainty

## Abstract

Optimization of spatial resource allocation is crucial for the successful control of invasive species under a limited budget but requires labor‐intensive surveys to estimate population parameters. In this study, we devised a novel framework for the spatially explicit optimization of capture effort allocation using state‐space population models from past capture records. We applied it to a control program for invasive snapping turtles to determine effort allocation strategies that minimize the population density over the whole area. We found that spatially heterogeneous density dependence and capture pressure limit the abundance of snapping turtles. Optimal effort allocation effectively improved the control effect, but the degree of improvement varied substantially depending on the total effort. The degree of improvement by the spatial optimization of allocation effort was only 3.21% when the total effort was maintained at the 2016 level. However, when the total effort was increased by two, four, and eight times, spatial optimization resulted in improvements of 4.65%, 8.33%, and 20.35%, respectively. To achieve the management goal for snapping turtles in our study area, increasing the current total effort by more than four times was necessary, in addition to optimizing the spatial effort. The snapping turtle population is expected to reach the target density one year after the optimal management strategy is implemented, and this rapid response can be explained by high population growth rate coupled with density‐dependent feedback regulation. Our results demonstrated that combining a state‐space model with optimization makes it possible to adaptively improve the management of invasive species and decision‐making. The method used in this study, based on removal records from an invasive management program, can be easily applied to monitoring data for wildlife and pest control management using traps in a variety of ecosystems.

## Introduction

Increases in populations of invasive species worldwide are degrading native ecosystems and negatively affecting human well‐being (Mack et al. 2000, Mooney and Hobbs, 2000, Butchart et al. 2010). Many noxious invasive species are widely distributed on large islands and continents where complete eradication is impossible (Lowe et al. 2000), requiring long‐term control (Grice 2009). To minimize damage by invasive species under limited budgets, it is necessary to quantify the resources available for control aimed at long‐term equilibrium outcomes (Perrings 2005). This requires an efficient and specific management plan based on scientifically sound evaluation methods (Byers et al. 2002).

Optimization of spatial resource allocation is a powerful tool for decision‐making in control programs for invasive species (Hauser and McCarthy 2009, Chadès et al. 2011, Giljohann et al. 2011, Epanchin‐Niell and Wilen 2012, Guillera‐Arroita et al. 2014, Jafari et al. 2018, Bonneau et al. 2019). This optimization is aimed at determining the allocation of management effort (costs, number of installed traps, labor, etc.) needed for each management unit to maximize the control outcome under a given budget (Adams and Setterfield 2015, Bonneau et al. 2017, Yemshanov et al. 2017). Optimal effort allocation is especially useful under environmental heterogeneity (Hauser and McCarthy 2009, Giljohann et al. 2011, Baker 2017), but it is often impractical to conduct detailed surveys for estimating population parameters.

However, time‐series data for exotic animal control (i.e., capture counts and capture effort) are useful for estimating population parameters (Fukasawa et al. 2013a, 2013b,*2013a*, *2013b*, Wells et al. 2016, Scroggie et al. 2018), including the natural growth rate, density effect on population growth, and capture efficiency. It should be noted that field‐based monitoring data are often limited by irregular spatial and temporal arrangements of traps and missing data, among other factors. In addition, process errors due to unknown environmental heterogeneity are also worrisome, making it difficult to understand precise ecological processes. (de Valpine and Hastings 2002). Considering process and observation errors simultaneously in statistical analyses is thus very important for estimating various parameters of population dynamics (Freckleton et al. 2006, Lebreton and Gimenez 2013).

State‐space models effectively separate process and observation errors (Valpine and Hastings 2002) and have been used to analyze various population dynamics involving capture pressure and environmental heterogeneity (Millar and Meyer 2000, Rivot et al. 2004, Fukasawa et al. 2013a, 2013b,*2013a*, *2013b*, Osada et al. 2019). Population growth rates often vary spatially (Clobert et al. 2009), thereby resulting in spatial correlations. Using the state‐space model to consider spatial correlation can reveal differences in population growth rates and carrying capacity, even with little information about environmental factors, providing a basis for the spatial optimization of control effort.

The common snapping turtle (*Chelydra serpentina*), which is widely distributed in freshwater ecosystems in North America (Steyermark et al. 2008), is an invasive species that poses a human injury risk as well as a threat to native ecosystems in Japan. In the Kashima River system in northern Chiba, the local government has conducted a removal program for snapping turtles since 2007 and a large number of traps have been set along the river system. Consequently, long‐term removal data (number of captures and capture effort) for each trap location are available for spatiotemporal analyses. The local government has also established a tentative management goal for the snapping turtle, defined as an annual CPUE (catch per unit effort; here, the number captured per number of trap‐days) <0.03, but this goal has not yet been achieved (Chiba Biodiversity Center 2016). Snapping turtle captures have a patchy spatial distribution, which may be affected by environmental heterogeneity. Because the budget for the removal program is limited, estimates of capture effort and effective spatial trapping arrangements to achieve the target density are urgently needed.

**Fig. 1:**
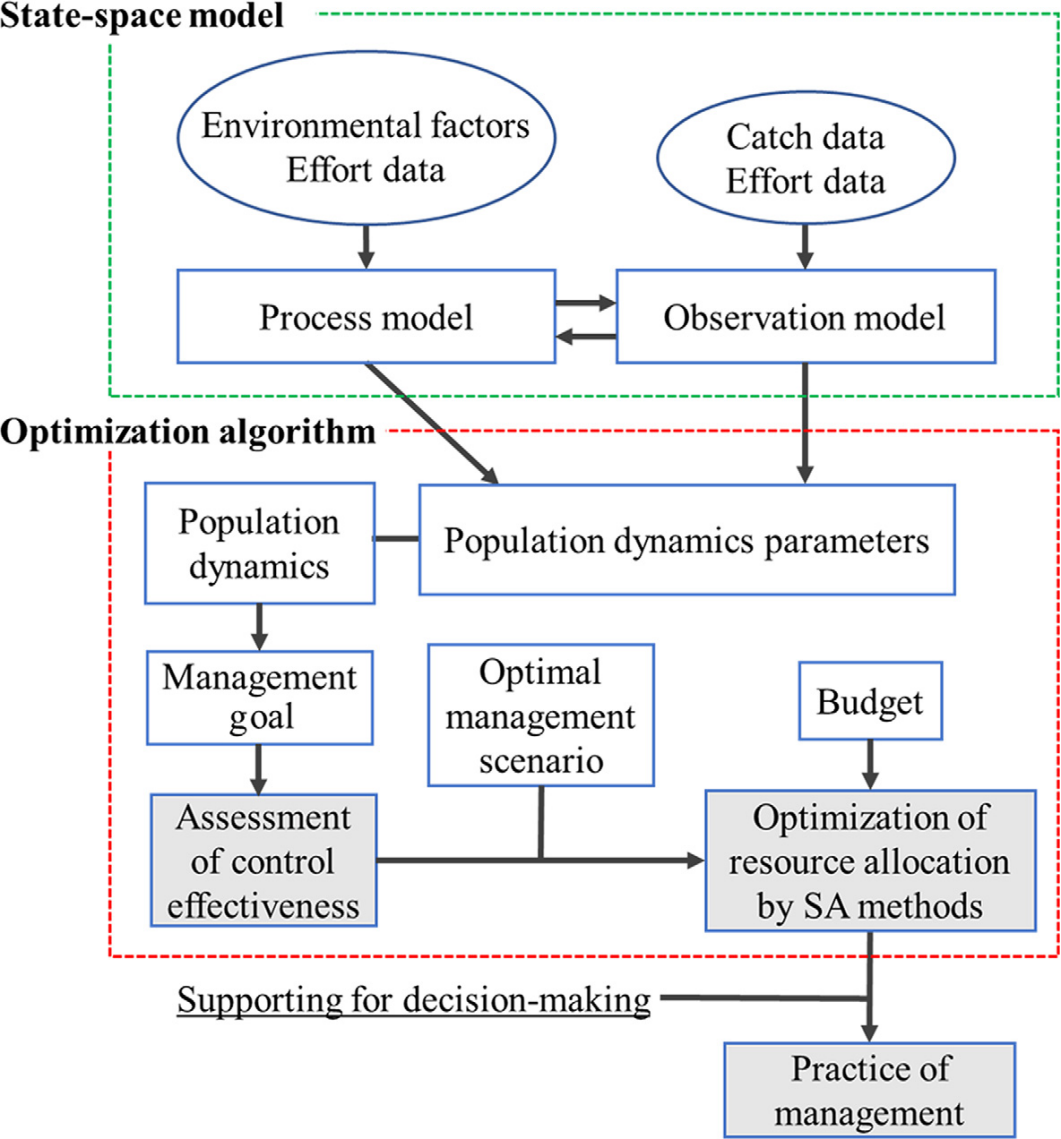
Workflow of the spatial optimization process using monitoring data. Removal records (catch data and effort data) from management practices can be used to estimate population dynamic parameters. Combining the state‐space model with spatial optimization makes it possible to estimate the population status and dynamics of target species and to evaluate control effectiveness, including the total effort needed to achieve management goals. The results contribute to decision‐making for the control of invasive species. SA, simulated annealing.

We devised a framework using invasive species removal records to estimate the population dynamics and optimize effort allocation, considering environmental heterogeneity and uncertainty (Fig. 1). The aim of this study was to develop an efficient spatial allocation strategy, assuming a fixed budget, to reduce the snapping turtle population to a density at which they no longer cause unacceptable damage. We developed a state‐space model describing the population dynamics of the snapping turtle in a spatially explicit manner. Similar to the model of Fukasawa et al. (2013*b*), we used CPUE (a density index) as a predictor variable, and local environmental factors, capture effort (trap‐days), and spatial autocorrelation as explanatory variables. We then optimized spatial effort allocation using a simulated annealing method, which minimized the sum of the equilibrium densities for the entire region. Finally, we estimated the effort needed to achieve the target density goal established by Chiba Prefecture.

## Materials and Methods

### Species and study area

The snapping turtle (*Chelydra serpentina*) is a freshwater turtle widely distributed from southern Canada to Texas in the southeastern United States (Steyermark et al. 2008). The species was exported from the United States to other countries as a commercial pet and food (Ceballos and Fitzgerald 2004, Schlaepfer et al. 2005) and was first imported to Japan in 1960 (Kobayashi et al. 2006). The first breeding in the wild was recorded in the Kashima river system in 2002 (Kobayashi et al. 2003). The snapping turtle was identified as a “Designated Invasive Alien Species” (prohibiting imports and restricting ownership and movement) in 2005 by the Ministry of the Environment owing to its potential influence on native ecosystems and human well‐being. This species is omnivorous with a strong carnivorous tendency (Ernst et al. 1994) and preys on other turtle species and conspecifics (Hammer 1969). In Japan, this species has a wide range of feeding habits, including aquatic plants, crustaceans, and fishes (Tsujii et al. 2012), and poses a threat to native ecosystems. Moreover, the turtles can injure humans, because the biting force of adults is very strong (Herrel et al. 2002). In fact, there are records of local fishermen and farmers suffering injuries from bites. There is an urgent need to eliminate the species to lower the risk of further damage.

**Fig. 2:**
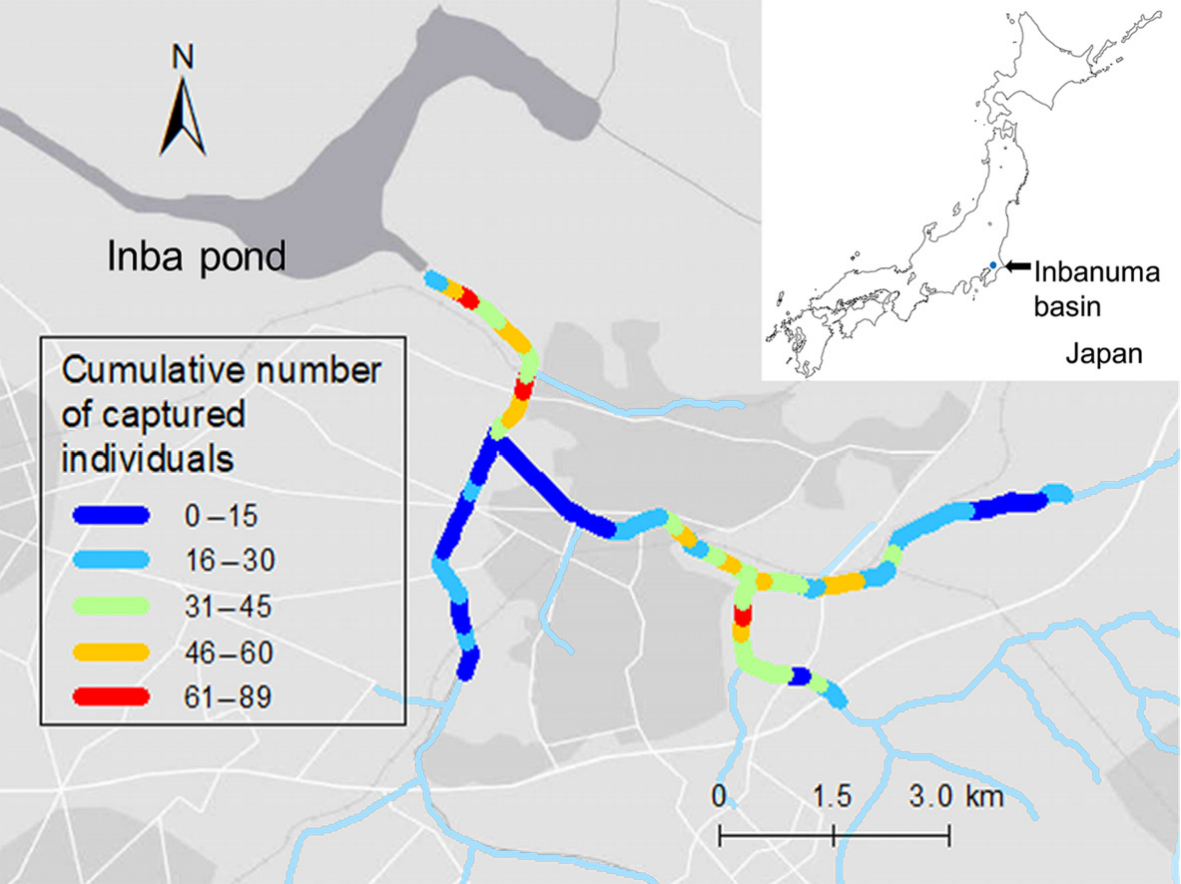
The Kashima River system in the Inbanuma Basin, Chiba, Japan and the cumulative number of captured individuals for snapping turtle control program. The light blue lines indicate rivers outside this study area. Background map sources: Esri, HERE, Garmin, OpenStreetMap contributors, and the GIS user community.

The study area was the Kashima River system (35°71′ N, 140°22′ E) in the Inbanuma basin located in northwestern Chiba Prefecture, Japan (Fig. 2). The average temperature in 2017 was 14.7°C and the lowest and highest temperatures in 2017 were −7.0°C and 34.2°C, respectively (Japan Meteorological Agency, data *available online*).^9^ Rice paddy field is the major land use type around the Kashima River system; the area has irrigation ditches and drainage waterways that provide habitats for snapping turtles. The snapping turtles in this area move from the river to the surrounding paddy fields and waterways (Kobayashi et al. 2006).

The Kashima River system is a high population density region of snapping turtles, and population management throughout the basin has been undertaken by the local government since 2008 after a small‐scale pilot removal program in 2007. Capture work is conducted from May to September, covering the whole active period, including the egg‐laying season. Traps baited with mackerel or bonito heads are set up at intervals of about 50–100 m along each river. The trap‐checking interval was generally every 2–3 d, and rarely every 4 d. In this region, long‐term removal data for each trap location are acquired by the local government because control has been performed continuously from the beginning of the removal project.

### General framework

The framework for the spatial optimization process consists of a series of flows of management practice, evaluation of control effectiveness, and optimization of effort allocation (Fig. 1). Routine removal records provide information on population dynamic parameters. Combining a state‐space model with spatial optimization enables the estimation of the population status and dynamics of target species and the evaluation of control effectiveness, such as total effort needed to achieve management goals.

### Data set

Trap data used for analyses were from 2008 to 2016. The trap data consisted of the total number of captures for each trap and trap‐days per year. Captured individuals were mostly subadults and adults. The river was divided into 75 200‐m sections that were used as units for analyses. The section size corresponds to approximately the maximum travel distance from the water body to the egg laying site by females, which is 183 m (Congdon et al. 1987).

In our analysis, five environmental factors were considered (paddy field area, dry crop fields, slope, road extension density, and flow accumulation). Paddy fields and dry crop fields are potentially suitable habitats for snapping turtles (Kobayashi et al. 2006, Thompson et al. 2017). Slopes are used as an index of snapping turtle egg laying sites (Takayama and Matsuzawa 2015). Road extension density is an indicator of the potential road‐kill risk (Gibbs and Shriver 2002). Flow accumulation was used as a quantitative indicator of river flow. Four environmental variables, excluding flow accumulation, were acquired from the National Land Numerical Information database (*available online* [in Japanese]),^10^ which provides information on land use (paddy field area and dry crop fields, 100‐m grid data; slope, elevation/slope 250‐m grid data; road extension density, road density/road extension 1‐km grid data), and flow accumulation was calculated using ArcGIS v. 10.5 (Environmental Systems Research Institute, Redlands, California, USA) from the 250‐m grid digital elevation model data downloaded from the above‐mentioned web site. These environmental factors were aggregated into the buffer of 200 m from the center line of each river section; areas within the buffer were calculated for paddy field and dry field, and average values were calculated for slope, road extension density and flow accumulation. The data set was prepared using ArcGIS v. 10.5.

### Model description and estimation

Bayesian state‐space models are used to analyze non‐Gaussian and nonlinear ecological processes from time‐series data (e.g., Knape and Valpine 2012, Fukasawa et al. 2013*b*; Hostetler and Chandler 2015). A state‐space model consists of a state process model and an observation process model. The state process, denoted as unobservable vector $x_{t}$ (*t* = 1, 2, …, *T*), represents a stochastic transition of ecological states. To analyze population dynamics, a first‐order Markov process, $x_{t}|x_{t‐1}$, is applied sequentially, and the initial state is defined by a random variable following a certain prior distribution. The observation process is an observable vector denoted $y_{t}$ related to $x_{t}$ that fluctuates with the observation error. These processes involve uncertainty and the Bayesian state‐space model is described as a hierarchical model using a set of three probability density functions (pdfs):

(1a) $$g_{t}\left(x_{t}|x_{t‐1};\eta \right)\;\text{state process pdf}$$

(1b) $$g_{1}\left(x_{1};\nu \right)\;\text{initial state pdf}$$

(1c) $$f_{t}\left(y_{t}|x_{t};\psi \right)\;\text{observation process pdf}$$

where η, ν, and ψ are vectors of model parameters.

In this study, the observation process represents the relationship between the number of captured individuals, **y** *_t_* = (*C*[1, *t*], *C*[2, *t*],..., *C*[75, *t*]), and the log relative density, **x** *_t_* = (*μ*[1, *t*], *μ*[2, *t*],..., *μ*[75, *t*]) in year *t* (*t* = 1, 2,..., 9) over the 9‐yr period from 2008 to 2016 in 75 spatial units. In this study, we assumed that CPUE was proportional to population density as commonly used in fishery stock management models. $E\left[i,t\right]$ denotes the capture effort (i.e., trap‐days), and expected value of CPUE, $E\left[C\left[i,t\right]/E\left[i,t\right]\right]$ was formulated by log relative density, $\mu \left[i,t\right]$ (Hilborn and Walters 2013, Thorson and Haltuch 2019)

(2) $$E\left[C\left[i,t\right]/E\left[i,t\right]\right]=\exp\left(\mu \left[i,t\right]\right).$$

As capture events are supposed to be mutually independent, the observed number of captures is assumed to follow a Poisson distribution with the mean value proportional to the product of $E\left[i,t\right]$ and the relative density, $\exp\left(\mu \left[i,t\right]\right)$

(3) $$C\left[i,t\right]∼\;\text{Poisson}\left(E\left[i,t\right]\exp\left(\mu \left[i,t\right]\right)\right).$$

In the state process, population dynamics over the range defined in space and time corresponding to the observation process are described by the stochastic Gompertz model (Dennis et al. 2006). This is a density‐dependent model assuming a linear autoregressive formulation of log population density $\mu \left[i,t+1\right]$, which is a linear model of the previous log population density $\mu \left[i,t\right]$. We considered the five environmental variables (i.e., paddy field area, dry crop fields, slope, road extension density, and flow accumulation, described above) and the capture effort in each section as linear predictors that potentially influence the population growth rate. The logarithm of the relative density of turtles $\mu_{i,t}$ is described as follows (Colchero et al. 2009, Fukaya et al. 2013, Fukasawa et al. 2013*b*):

(4) $$\mu \left[i,t+1\right]=\alpha +\lambda \mu \left[i,t\right]+\sum \beta_{k}H_{k}\left[i\right]+\beta_{6}E\left[i,t\right]+\rho \left[i\right]+\omega \left[t\right]+e\left[i,t\right]$$

where α is an intercept and λ is the density dependence on the logarithmic relative density in the next year. Density dependence is absent when = λ1. $\beta_{k}$ (k=1,2,⋯,5) are parameters for environmental factors $H_{k}\left[i\right]$ (paddy field area, dry crop fields, slope, flow accumulation, and road extension density). All of these environmental factors were standardized to average 0 and standard deviation (SD) 1 so that the coefficients can be interpreted as changes in the log(population growth rate) with + 1 SD of factors. $\beta_{6}$ represents the effect of capture effort E[i,t] (scaled by 100 trap‐days). With reference to Fukasawa et al. 2013*b*, parameters of process error are described below. The parameters $\omega \left[t\right]$ and e[i,t] are annual variation in the population growth rate for the entire study area and random noise of the population growth rate; they follow a normal distribution with mean 0 and variances $\sigma_{\omega }^{2}$ and $\sigma_{e}^{2}$, respectively

(5) $$\omega \left[t\right]∼\text{Normal}\left(0,\sigma_{\omega }^{2}\right)$$

(6) $$e\left[i,t\right]∼\text{Normal}\left(0,\sigma_{e}^{2}\right).$$

The spatially structured variation $\rho \left[i\right]$ indicating spatial trends in growth rates due to unmeasured environmental factors and dispersal processes is expressed by the Proper CAR prior (Latimer et al. 2006)

(7) $$\rho \left[i\right]|\rho \left[‐i\right]∼N\left(\frac{\sum_{j\in \delta \left[i\right]}\gamma \rho \left[j\right]}{k\left[i\right]},\frac{\sigma_{\rho }^{2}}{k\left[i\right]}\right)$$

where $\rho \left[‐i\right]$ is all elements of ρ except $\rho \left[i\right]$. $\delta \left[i\right]$ and $k\left[i\right]$ are a class of neighbors defined as units sharing the same edge of *i* and their number, respectively. The parameter γ controls the overall strength of the spatial dependence and $\sigma_{\rho }^{2}$ is the conditional variance related to the spatial divergence among neighboring units. By incorporating spatial autocorrelation into the model, it is possible to estimate uncertainty appropriately and to improve the prediction accuracy of the model (Latimer et al. 2006, Sims et al. 2008).

For the initial state pdf (i.e., prior distribution of the initial state), the logarithmic relative density in the first year follows a normal distribution with mean 0 and variance $\sigma_{1}^{2}$, which represents the variance of the initial state among units

(8) $$\mu \left[i,1\right]∼\text{Normal}\left(0,\sigma_{1}^{2}\right).$$

Regarding the prior distributions, $\sigma_{\omega }^{2}$ and $\sigma_{1}^{2}$ follow a weakly informative prior distribution (i.e., a half‐normal distribution with mean 0 and variance 100). A non‐informative prior (flat prior) was used for other parameters.

Sampling from posterior distributions was performed with the Markov chain Monte Carlo (MCMC) method using Stan v. 2.17.3, which employs the no‐u turn sampling algorithm (Hoffman and Gelman 2014, Carpenter et al. 2017, Stan Development Team 2018). To eliminate the influence of the initial value and autocorrelation in MCMC samples, the number of MCMC iterations was set to a sufficiently large value (iterations = 10,000) and results were extracted every four iterations after discarding the first 5,000 iterations. This calculation was performed for four chains with different initial values, and the posterior distribution was obtained. The convergence of MCMC sampling was assessed using $\hat{R}$ (Gelman and Rubin 1992), and the convergence criterion was set to $\hat{R}$ < 1.1. To check adequacy of model fit to the data, we conducted posterior predictive checks by comparing observed total captured numbers per year and the corresponding posterior predictions. Bayesian *P*‐value (Gelman et al. 2013) was calculated to evaluate discrepancy between observations and posterior predictions.

The relative contribution of the fixed effect was estimated to investigate whether unknown environmental factors influence population growth (Silber et al. 1995). The following equation was used:

(9) $$\delta =\;V1/\left(V1+\;V2\right)$$

where V1 and V2 are the variances of $\sum \beta_{k}H_{k}\left[i\right]$ (environmental factors) and $\rho \left[i\right]$ (spatial random effect), respectively, calculated from the MCMC sample.

### Optimization of spatial effort allocation

We conducted optimization of spatial effort allocation that minimize equilibrium population density over all spatial units under the static removal strategy (i.e., capture effort allocation is not changed with time). In the Gompertz model, when |λ| < 1 (λ: density dependence parameter, see Eq. 4), the average relative density eventually converged to the equilibrium density. The carrying capacity (*K*) was obtained by summing the equilibrium densities over all units

(10) $$K\left(E\right)=\sum_{i}\exp\left(\frac{\left(\alpha +\sum \beta_{k}H_{k}\left[i\right]+\beta_{6}E\left[i\right]+\rho \left[i\right]\right)}{\left(1‐\lambda \right)}\right).$$

The objective function $U\left(E\right)$ is the equilibrium density given effort for each area and is proportional to the number of individuals in the whole area. The equilibrium density (logarithmic value) itself will also have uncertainty, and this was accounted for in the optimization of management effort allocation. The expected value of the total density averaged over all MCMC samples was defined as the objective function. The objective function with effort as a variable is now defined by

(11) $$U\left(E\right)=\frac{1}{5000}\left(\sum K{\left(E\right)}_{n}\right).$$

Here, *n* is the MCMC sample number (1,2,3,⋯,5,000). The total capture effort $E_{\mathrm{total}}$ is given and Eq. 11 is a constrained optimization problem with $\sum E\left[i\right]=E_{\mathrm{total}}$ as a constraint.

A multivariate optimization approach was used by searching for the spatial effort allocation that minimizes *U* (**E**). This concept is similar to those of earlier studies exploring efficient spatial effort allocation to minimize the density of invasive species (e.g., Taylor and Hastings 2004, Epanchin‐Niell and Hastings 2010, Giljohann et al. 2011, Epanchin‐Niell and Wilen 2012, Baker 2017, Yemshanov et al. 2017). To convert an *I*‐dimensional constrained optimization problem to an unconstrained optimization problem of *I* – 1 dimensions, we introduced auxiliary variables $a=\left(a\left[1\right],a\left[2\right],\cdots ,a\left[I‐1\right]\right)$ and E[i] reparameterized as follows:

(12) $$E\left[i\right]=\left\{\begin{array}{c} E_{\text{total}}\frac{\exp\left(a\left[i\right]\right)}{1+\sum \left(\exp\left(a\left[i\right]\right)\right)},i\neq I\\ E_{\text{total}}\frac{1}{1+\sum \left(\exp\left(a\left[i\right]\right)\right)},i=I. \end{array}\right)$$

The optimal set of **a** was searched using a simulated annealing (Kirkpatrick et al. 1983, hereinafter referred to as the SA), an optimization algorithm with stochastic fluctuation to find a solution to minimize an objective function. The GenSA package (Xiang et al. 2013) of R (v. 3.5) was used for SA. Although results of effort allocations by SA are just an approximate optimal solution, we used the term “optimal” for them in this paper for simplicity. R code and data for optimal spatial allocation of capture effort can be found in Data S1.

To evaluate the effect of the total amount of effort on the spatial pattern of optimal effort allocation and improvement of population control by optimization, we evaluated the optimal allocation under different values for $E_{\mathrm{total}}$(1 to 8 times the total effort in 2016). We compared the performance of the optimal effort allocation to the actual effort allocation in 2016 and uniform allocation assuming the same amount of effort in each unit. The degree of improvement by optimization was calculated from the ratio of the equilibrium density between different scenarios, such as 1 − *U*(*E*_optimal_)/*U*(*E*_2016_) and 1 − *U*(*E*_optimal_)/*U*(*E*_uniform_).

### Simulation of the time to reach an equilibrium density

The time required to reach an equilibrium density in our management scenarios was evaluated by a simulation approach based on MCMC sample parameters estimated by the state‐space model. As optimal resource allocation in the equilibrium state is not always optimal in the non‐equilibrium state, we investigated performance in a dynamic process based on the state of convergence. This simulation was performed using R under optimal effort allocation, which minimizes the equilibrium density by using the process model with stochasticity with posterior samples (Eq. 4). Temporally structured variation $\omega \left[t\right]$ and unstructured process variation e[i,t] were generated from probability distributions; they followed a normal distribution with mean 0 and variance estimated parameters $\sigma_{\omega }^{2}$ and $\sigma_{e}^{2}$, respectively.

## Results

**Fig. 3:**
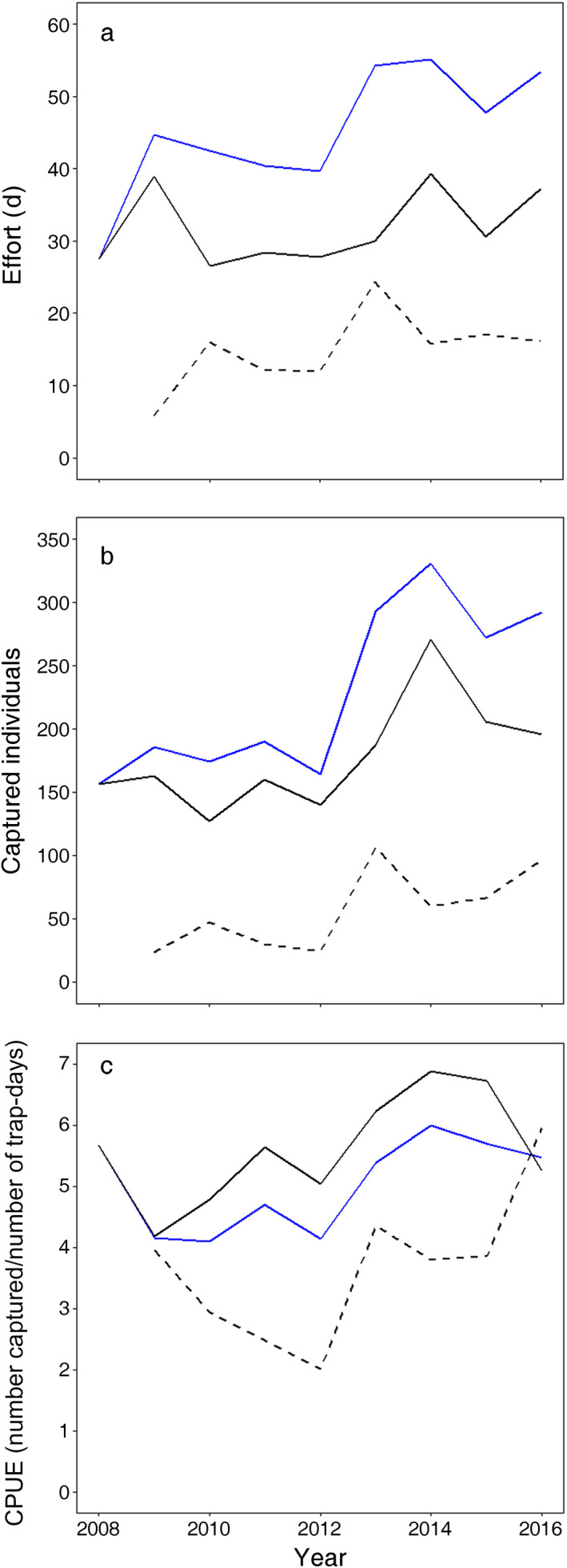
(a) Effort amount (number days out of 100 trap‐days) and (b) captured individuals for each year, and (c) catch per unit effort (CPUE) in the study area (number captured per number of trap‐days). The blue solid line indicates the total value in whole study area. The black solid line and dashed line indicate the total value in the control area in 2008 (41 sites) and in area except where it was managed in 2008, respectively (34 sites).

During the period 2008–2016, CPUE for the entire region increased slightly despite an overall increase in total effort (Fig. 3). The increase in total effort was due to an increase in the allocation of effort to peripheral regions in the controlled area not in areas previously controlled. The spatial effort allocation within controlled area varied among years (Appendix S1: Fig. S1). The densities in areas where the control program had started earlier were generally higher than those at the other areas except in 2016.

**Fig. 4:**
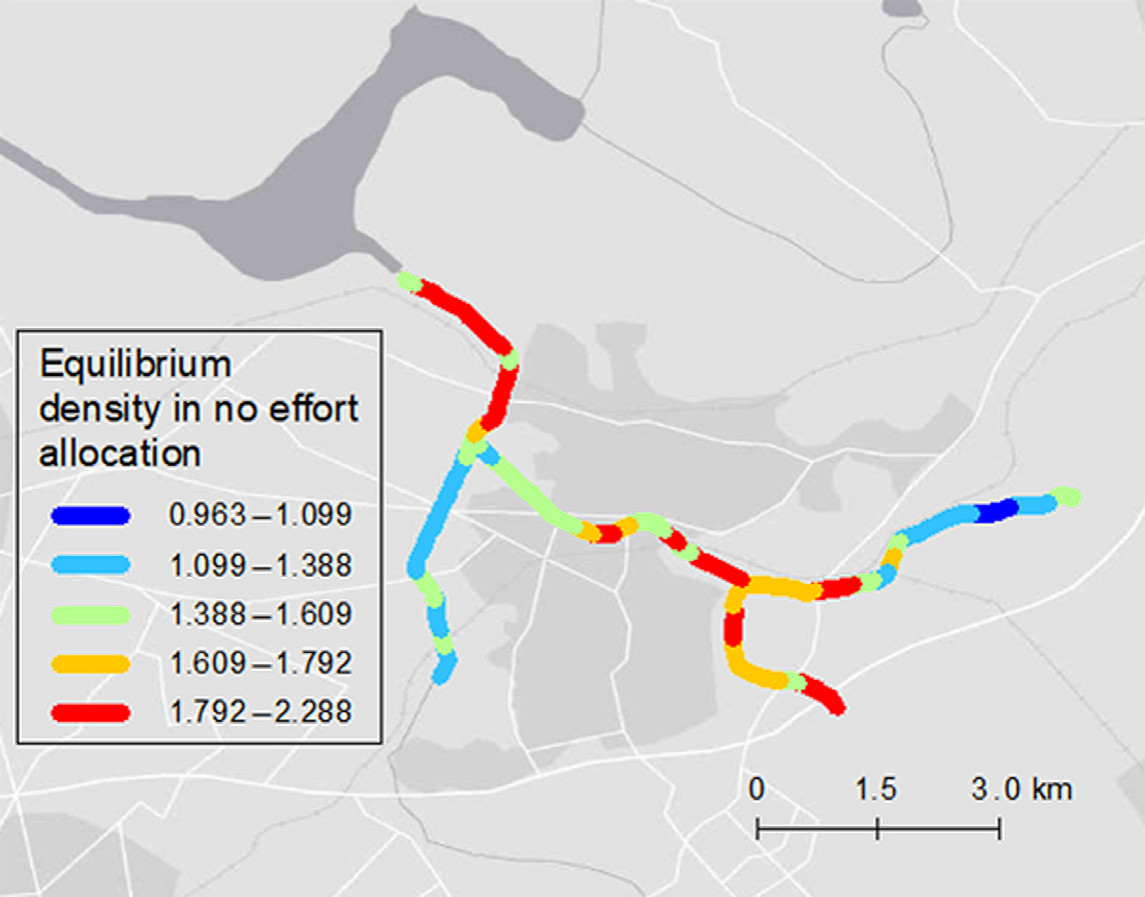
The equilibrium state without capture effort (carrying capacity). Background map source: Esri, HERE, Garmin, OpenStreetMap contributors, and the GIS user community.

We successfully estimated a range of parameters related to population dynamics using a state‐space model with spatial autocorrelation, incorporating environmental factors and effort. MCMC chain convergence was obtained, as evidenced by the Gelman‐Rubin statistic ($\hat{R}$) of < 1.1. In comparing the posterior prediction of total numbers captured per year with actual data, our dataset was not an extreme sample from the posterior prediction (the range of Bayesian *P* values for annual captured individuals each year were 0.311–0.714, Appendix S2: Table. S1). Table 1 shows the posterior summary of each parameter estimated by the MCMC method. The 95% CI of the density dependence parameter λ fell within 1 and −1, indicating that the study system converges to an equilibrium state based on the characteristics of the Gompertz model. The convergence was accompanied by oscillation because the upper limit of the 90% CI was negative. The 95% CI of the removal effort parameter $\beta_{6}$ was less than 0 and the posterior samples did not include any positive values. For environmental factors, the 80% CI of the slope parameter was included in the positive range. Although none of the environmental factors strongly influenced demography, spatial random effects indicated that there was clear spatial variation in the population growth rate and carrying capacity. The median relative contribution of the fixed effect against the spatial random effect was 0.218 (95% CI 0.053–0.419), indicating that the spatial random effect strongly influenced heterogeneity in the carrying capacity (Fig. 4).

**Table 1:** Summary of the posterior parameters (posterior mean, standard deviation of Markov chain Monte Carlo [MCMC] samples, 80% and 95% credible interval, CI).

Parameter	Mean	SD	2.5% CI	10% CI	90% CI	97.5% CI
alpha (intercept)	1.868	0.191	1.508	1.627	2.111	2.250
lambda (density dependence)	−0.159	0.087	−0.329	−0.271	−0.048	0.012
beta1 (paddy)	−0.054	0.126	−0.301	−0.218	0.102	0.195
beta2 (dry crop fields)	−0.042	0.104	−0.234	−0.172	0.091	0.170
beta3 (slope)	0.182	0.122	−0.060	0.025	0.332	0.417
beta4 (flow accumulation)	0.111	0.111	−0.112	−0.033	0.249	0.328
beta5 (road)	0.008	0.131	−0.253	−0.161	0.176	0.260
beta6 (effort)	−0.262	0.083	−0.426	−0.369	−0.156	−0.096

**Fig. 5:**
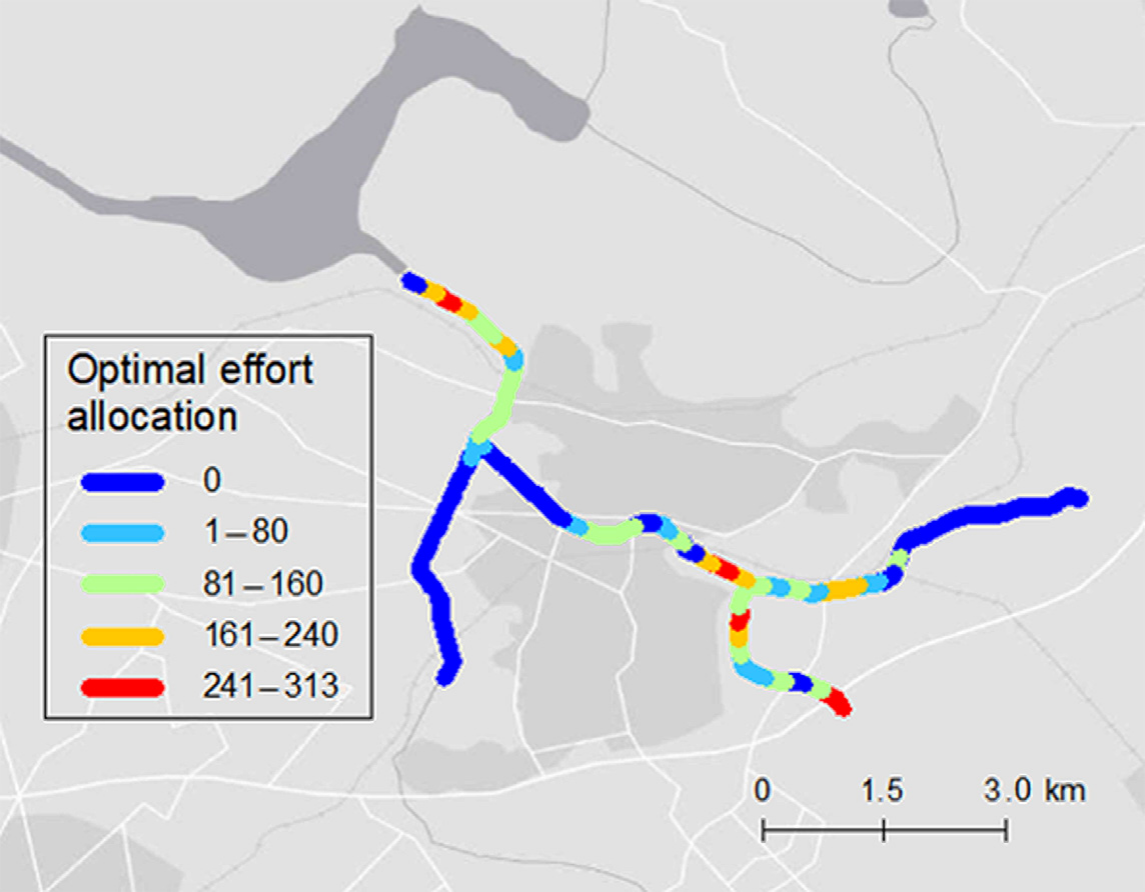
Maps showing the optimal effort allocation (trap‐days) calculated by the simulated annealing (SA) method. Total amount of effort is the same as actual effort in 2016. Background map source: Esri, HERE, Garmin, OpenStreetMap contributors, and the GIS user community.

**Fig. 6:**
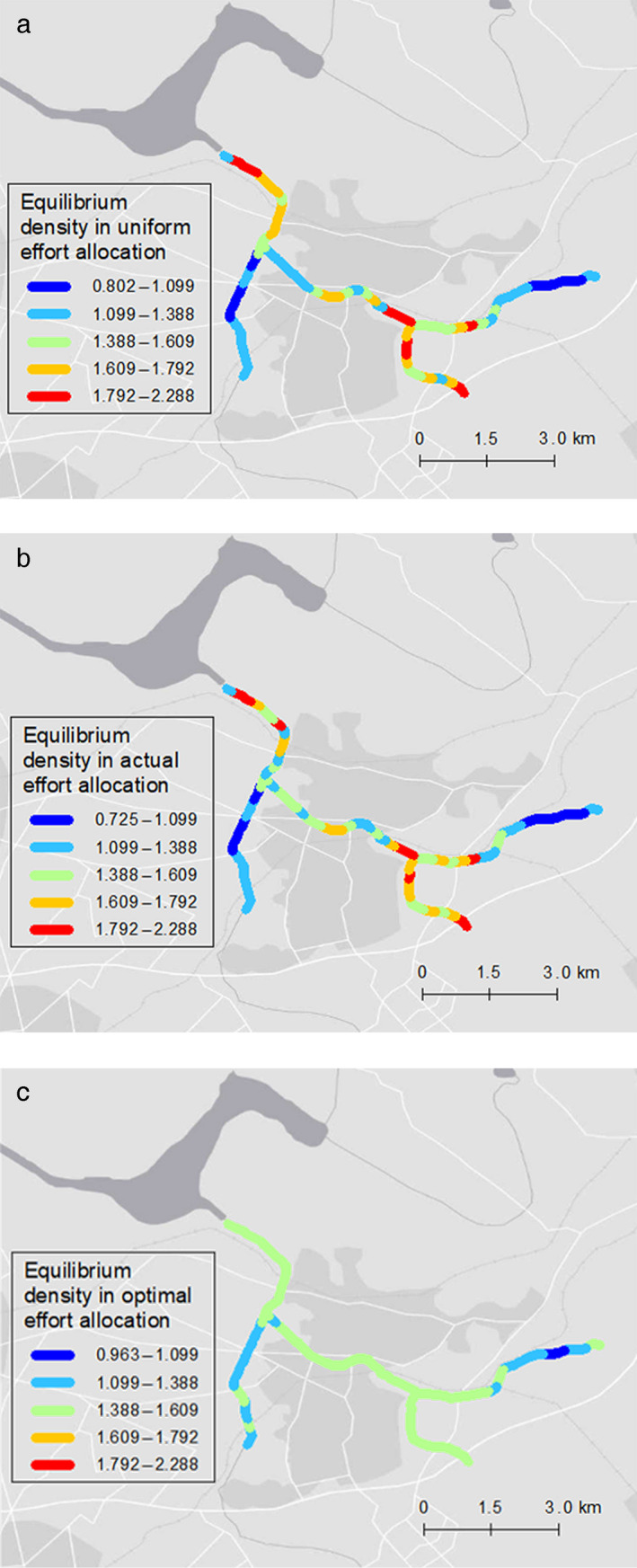
Maps showing equilibrium states for different allocation scenarios. (a) Uniform effort allocation, (b) effort allocation in the latest year (2016), (c) optimal effort allocation calculated by the simulated annealing method. Total amount of effort for panels a–c is the same as the actual effort in 2016. Background map source: Esri, HERE, Garmin, OpenStreetMap contributors, and the GIS user community.

Spatially optimal effort allocation was evaluated, considering spatial heterogeneity influencing the population dynamics of snapping turtles (Fig. 5). Optimal effort showed a more clumped distribution than the actual effort distribution in the latest year (Figs. 5 and Appendix S1: Fig. S1(b9)). We also obtained density maps of the equilibrium relative population density under three different management scenarios, holding the total effort in 2016 constant: uniform effort allocation, actual effort allocation, and optimal effort allocation (Fig. 6). The spatial pattern of optimal removal effort was concentrated in areas where the equilibrium density and the cumulative number of captures were high (Figs. 2, 4, and 5).

The degree of improvement by the optimization of effort allocation, as expressed by density suppression, depended on the total amount of effort (Table 2). Compared with the spatially uniform effort, the control effect by optimization improved by 3.13%–3.97% when we changed the total amount of effort. The improvement by optimization relative to uniform allocation was maximized at an intermediate amount of total effort. Compared with uniform effort allocation, the degree of improvement by optimization increased when the total amount of effort increased. When the total effort increased by two, four, and eight times that in 2016, optimization of the effort distribution resulted in improvements of 4.65%, 8.33%, and 20.35%, respectively. Simulation experiments revealed that the population density in the entire region reaches an equilibrium state immediately, regardless of the total amount of effort and the allocation scenario (Appendix S3: Fig. S1).

**Table 2:** Values of objective function and mean relative densities under various total effort and effort allocation scenarios.

	Objective function			Mean relative density			Improvement (%)	
Multiplier of effort	UA	AA	OA	UA	AA	OA	OA vs. UA	OA vs. AA
1	341.0381	340.573	329.656	0.045	0.045	0.044	3.337	3.205
2	290.6886	292.809	279.192	0.039	0.039	0.037	3.955	4.650
3	248.4459	254.466	238.592	0.033	0.034	0.032	3.966	6.238
4	212.9157	223.258	204.666	0.028	0.030	0.027	3.875	8.328
5	182.957	197.583	176.197	0.024	0.026	0.023	3.695	10.824
6	157.6337	176.271	152.113	0.021	0.024	0.020	3.502	13.705
7	136.1766	158.447	131.662	0.018	0.021	0.018	3.316	16.905
8	117.9513	143.441	114.254	0.016	0.019	0.015	3.134	20.347

Notes

Uniform allocation (UA) indicates that averaged removal effort allocation is maintained. Actual allocation (AA) indicates that removal effort allocation in 2016 is maintained. Optimal allocation (OA) indicates that optimal spatial allocation calculated by simulated annealing is maintained. “Multiplier of effort” indicates the multiplier of the total amount of effort in 2016. Objective function gives the equilibrium density in each allocation scenario. Relative density reports the mean relative density in each allocation scenario. Improvement is the percent improvement between two allocation scenarios.

## Discussion

We developed a method for optimizing spatial resource allocation for exotic species control in spatially heterogeneous environments based on a Bayesian state‐space model with actual observation data. This approach has two major methodological advances compared with earlier studies that addressed similar issues. First, this method has relaxed design constraints for estimating parameter values that are required for the optimization of effort allocation. Conventional catch‐effort analyses require data collected over a short period to satisfy the closed population assumption, with no recruitment or mortality during the sampling period (e.g., Chee and Wintle 2010, Rout et al. 2018). Our approach can be applied to more realistic situations in invasive species control programs, typically involving continuous removal, with reproduction and death occurring during the control period. Second, our method can estimate population dynamics and spatial optimization in spatially heterogeneous environments for species with incomplete data structures, including observation errors and missing values, unlike in earlier studies (Hauser and McCarthy 2009, Giljohann et al. 2011, Baker 2017). As invasive animal control is common and removal records are routinely obtained (Jones et al. 2016), our method can be applied to various situations and can therefore contribute to optimal decision‐making.

Our analysis revealed several determinants of snapping turtle population dynamics, including density dependence, local slope, and anthropogenic removal (Table 1). The coefficient for density dependence was negative, indicating that the population density was highly regulated in this system. This result contradicts the findings of a long‐term cohort study in Ontario, Canada, which showed little negative density dependence in the snapping turtle population (Brooks et al. 1991). The negative density dependence detected in our study could be explained by a lower mortality rate in our study area, higher density due to the absence of predators (e.g., otters and foxes; Kobayashi et al. 2006), and stronger bottom‐up effects. Also, snapping turtles in the study frequently immigrate from adjacent areas (paddy fields and waterways) to rivers (Kobayashi et al. 2006), causing apparent density dependence at low densities. With respect to environmental variables, the slope surrounding the river positively influenced the density. This is in agreement with local knowledge that the snapping turtle tends to select sloping lands facing water bodies for egg laying (Takayama and Matsuzawa 2015).

Our approach can be used not only to search for the most effective control strategy but also to evaluate the system response to anthropogenic removal. The relative density in areas where the control program had started earlier did not apparently decrease despite the continuous removal (Fig. 3). We considered that, due to strong density‐dependent population regulation mentioned above, our study system would reach equilibrium population density immediately under given removal effort and did not show a constant population decline by anthropogenic removal (Appendix S3: Fig. S1), in contrast to other invasive species management, such as in a mongoose control program (Fukasawa et al. 2013*a*).

The optimized resource allocation scenario outperformed the actual and uniform effort allocation scenarios with respect to decreasing the equilibrium population density of snapping turtles, and the benefit of optimization depended on total amount of effort (Fig. 6, Table 2). A unimodal pattern of improvement by optimization against uniform effort allocation would be due to a nonlinear relationship between the total capture effort and equilibrium density. The decrease in density will be small, irrespective of the allocation strategy, if the total effort is low, and therefore the results will not be affected by resource allocation strategies. When the total amount of effort is sufficiently large, the density approaches 0 everywhere. Thus, compared to the uniform resource allocation scenarios, the degree of improvement by the spatial optimization was greater when the total effort amount was moderate. Our method can be used to estimate the cost to achieve a management goal, defined as a target population density. For example, it is necessary to increase total control effort by at least 4 times the current one in order to ensure the target density level of snapping turtle is met (i.e., CPUE < 0.03, defined by the management plan in Chiba Prefecture [Chiba Biodiversity Center 2016] Table 2). In invasive species management, management goals may change in response to new information about costs and benefits of management (Buckley 2008). Our results on optimal effort allocation will be also valuable when the management target is reevaluated in the future.

The optimal effort allocation strategy described in this study can be summarized as “allocating control effort to the highest density areas” (Figs. 2, 4, and 5). This optimal strategy was also identified in the previous studies, which explicitly modeled diffusion processes of invasion (Baker 2017, Bonneau et al. 2017) except for immediately after invasion (Baker 2017). For controlling established invasive species, an optimized strategy would be a primary choice for managers.

The effort allocation based on a greedy algorithm (Cormen et al. 2009) that gives priority to the unit with the highest predicted equilibrium density was almost the same to the optimal allocation based on the SA method (Appendix S4: Table S1 and Fig. S1). This was because the objective function was the sum of equilibrium densities over spatial units and equilibrium density of each unit is a convex function of effort (i.e., exponential with a negative coefficient of effort). In such a situation, greedy algorithms can find optimal solutions (Federgruen and Groenevelt 1986), and it can be a simpler option than the SA method.

Our study provides a basis for the implementation of the optimization approach being linked to adaptive management based on accumulated removal records for decision‐making. An adaptive or iterative process of “learning by doing” (Walters and Holling 1990) is compatible with Bayesian updating, which improves estimation accuracy. As managers learn more about systems through accumulated data related to management effort and its consequences, they can improve estimation accuracy and optimize spatial effort allocation. Although adaptive management has been developed in earlier studies, few applications to actual invasive species management have been reported (Westgate et al. 2013). This may be due to the lack of an established method for adaptive management. Our study provides a practical approach for field management. Although we did not perform iterative optimization, it is possible to update the spatially optimal resource allocation sequentially during continuous monitoring, as in previous studies (e.g., Hauser and McCarthy 2009, Giljohann et al. 2011, Hauser et al. 2016, Baker et al. 2018, Rout et al. 2018).

By combining the state‐space model and optimization method, we optimized spatial resource allocation based on knowledge gained from the implementation of invasive species management programs. Although we focused on the population that has invaded the study site, we did not consider the diffusion process. However, state‐space models can be extended to estimate diffusion‐reaction equations (Wikle 2003) for spreading invasive species. State‐space models incorporating observation errors have been applied in various fields of wildlife management (Royle and Dorazio 2008, Yamamura et al. 2008, Iijima et al. 2013, Osada et al. 2015, Iijima and Ueno 2016) and in agricultural pest control (Yamamura et al. 2006, Fabre et al. 2010, Skevas and Serra 2016). The method presented here can be applied to wildlife management and pest control and has broad implications for these fields. For the control of invasive organisms, it is important to consider community‐level effects, not just effects on individual species. It is necessary to evaluate the impact of the relaxation of a top‐down trophic cascade induced by the removal of invasive predators. Because mesopredator release occasionally occurs by the removal of top predators (Crooks and Soulé 1999), the careful monitoring of invasive species other than the target invasive species will be necessary. Analyses of population dynamics and the optimization of resource allocation based on the state‐space model presented here will be a useful tool in such a community context.

## Supporting information

Appendix S1

Appendix S2

Appendix S3

Appendix S4

Data S1

## Footnotes

^9^
www.jma.go.jp/jma/index.html

^10^
https://nlftp.mlit.go.jp/ksj/index.html
