# Supplementary material for: Spatial optimization of invasive species control informed by management practices
Source: Ecol Appl. 2021 Jan 21;31(3):e02261. doi: 10.1002/eap.2261 (PMC8047888; doi:10.1002/eap.2261)
Supplement: Supplementary file 1 — Appendix S1 [file EAP-31-e02261-s005.pdf]

Supporting Information. Nishimoto, M., T. Miyashita, H. Yokomizo, H. Matsuda, T. Imazu, H. Takahashi, M. Hasegawa, and K. Fukasawa. 2020. Spatial optimization of invasive species control informed by management practices. Ecological Applications.

Appendix S1. Spatial allocation in capture effort for snapping turtle control program and spatial distribution of snapping turtles

We visualized change in the distribution of capture effort over time and snapping turtles (Fig. S1). The maps showed actual effort allocation (100 trapping days) for (a1-9) 2008 to 2016 and the median relative density of snapping turtles for (b1-9) 2008 to 2016. We used ArcGIS v 10.5 (Environmental Systems Research Institute, Redlands, CA) for visualization of spatial distribution patterns.

Fig. S1 (a1)

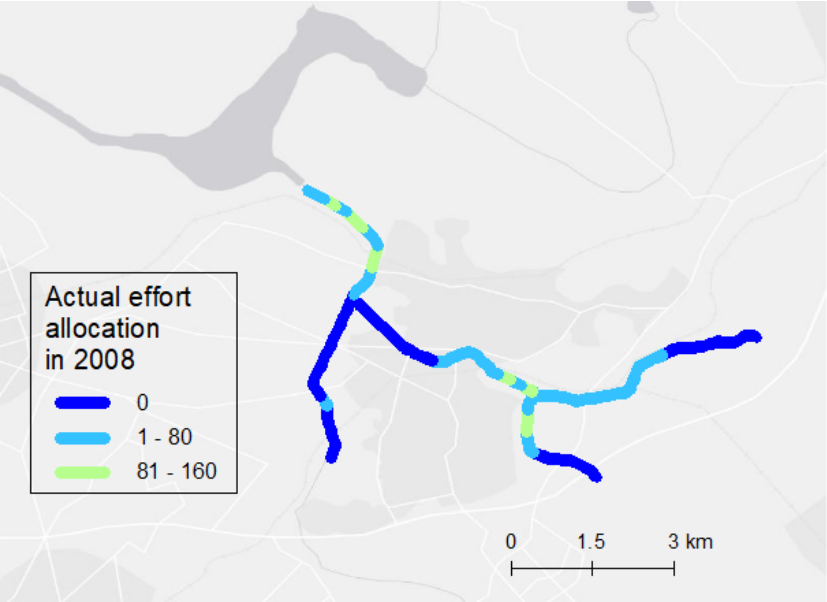

Fig. S1 (a2)

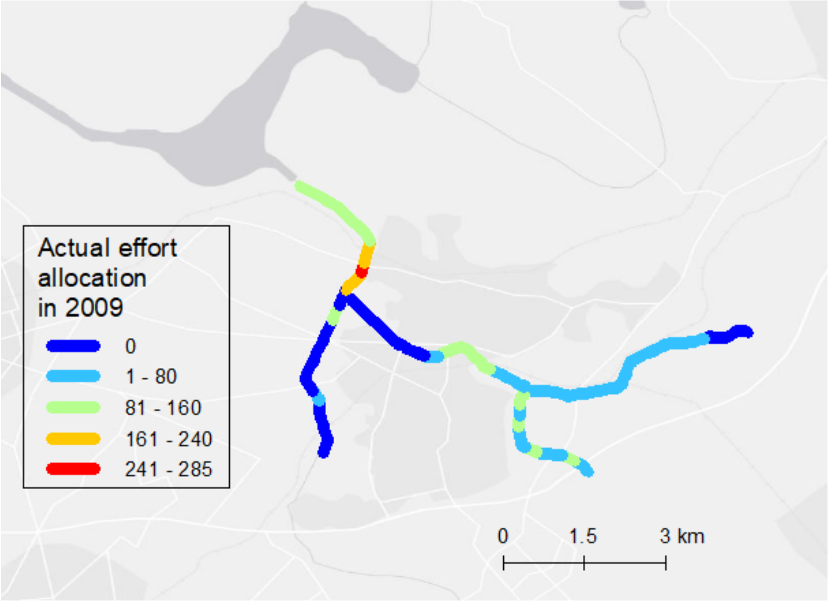

Fig. S1 (a3)

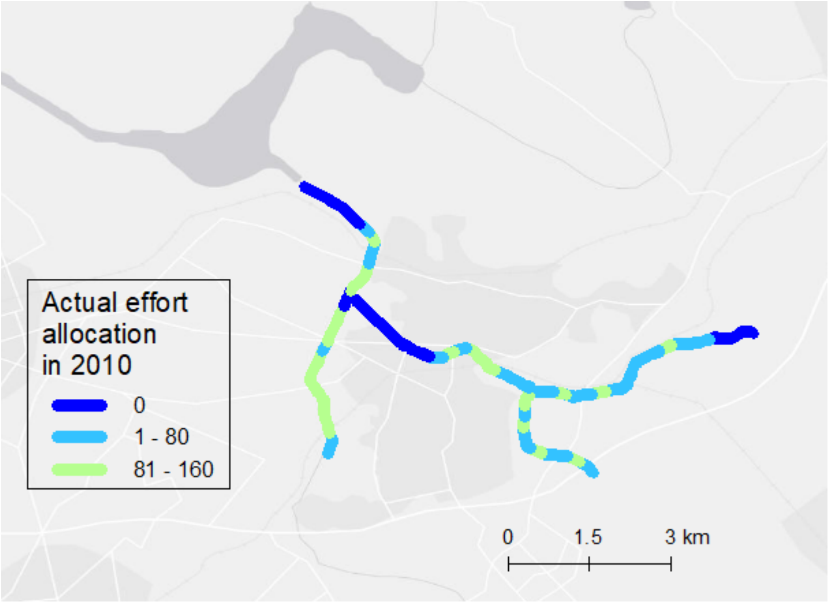

Fig. S1 (a4)

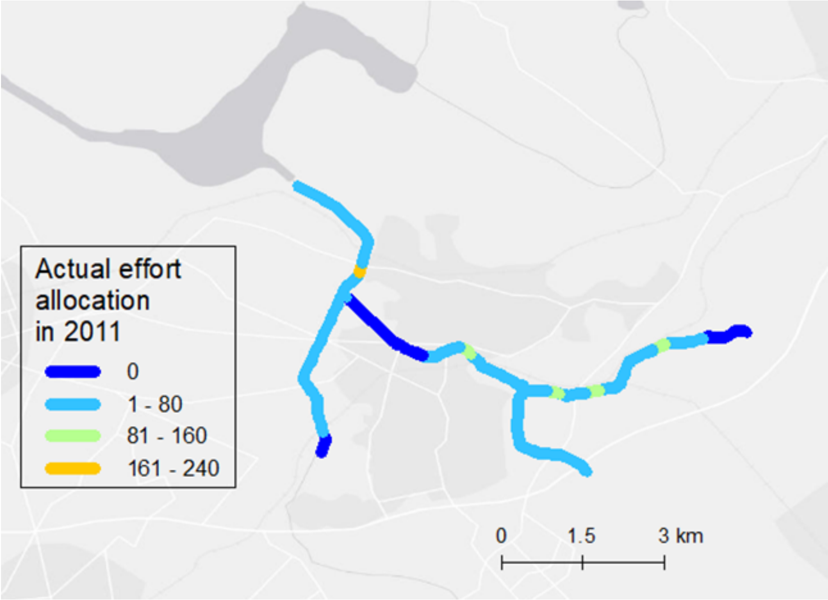

Fig. S1 (a5)

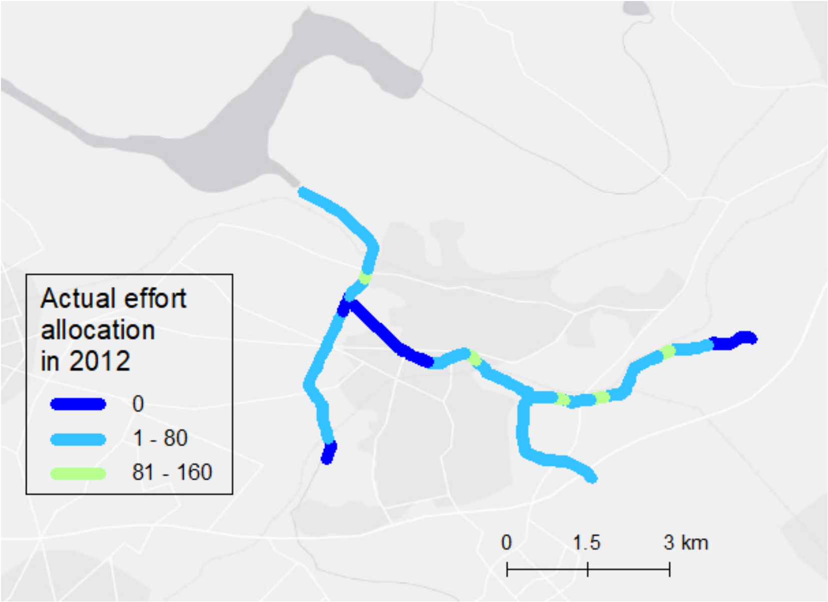

Fig. S1 (a6)

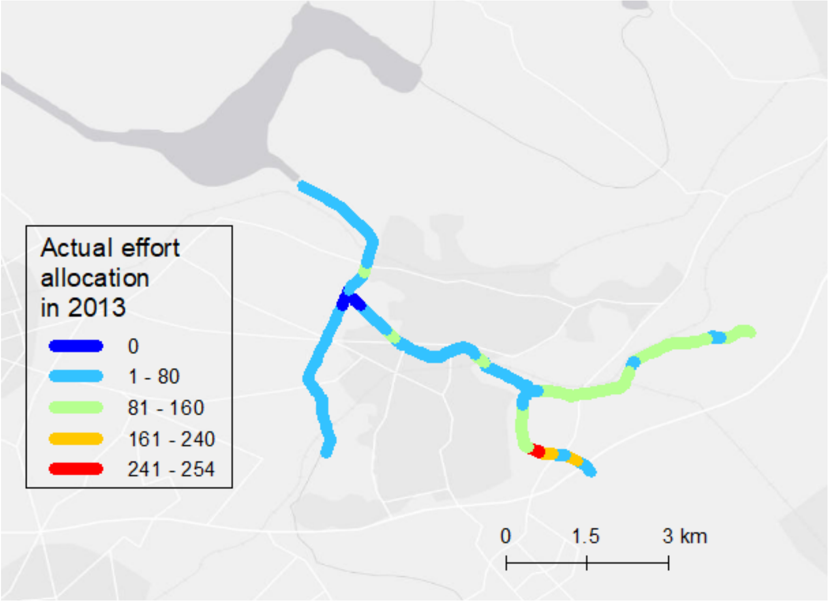

Fig. S1 (a7)

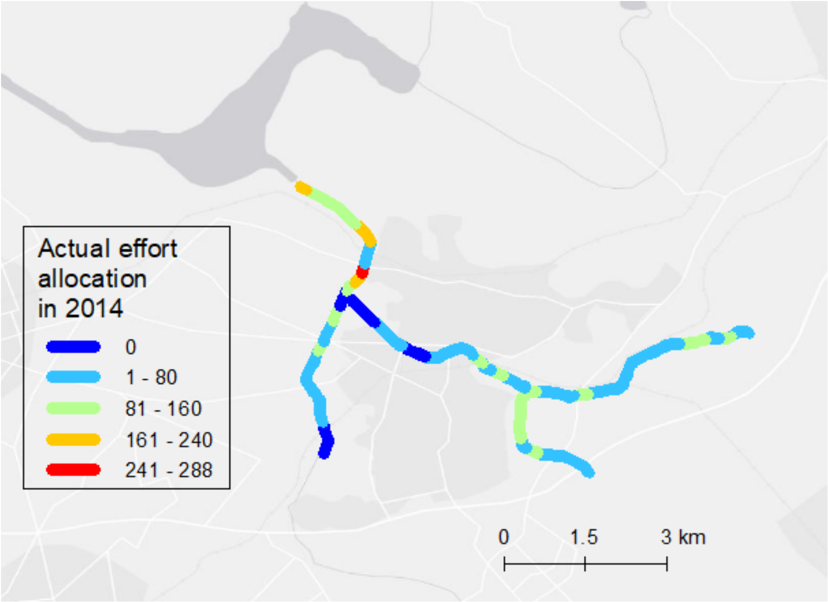

Fig. S1 (a8)

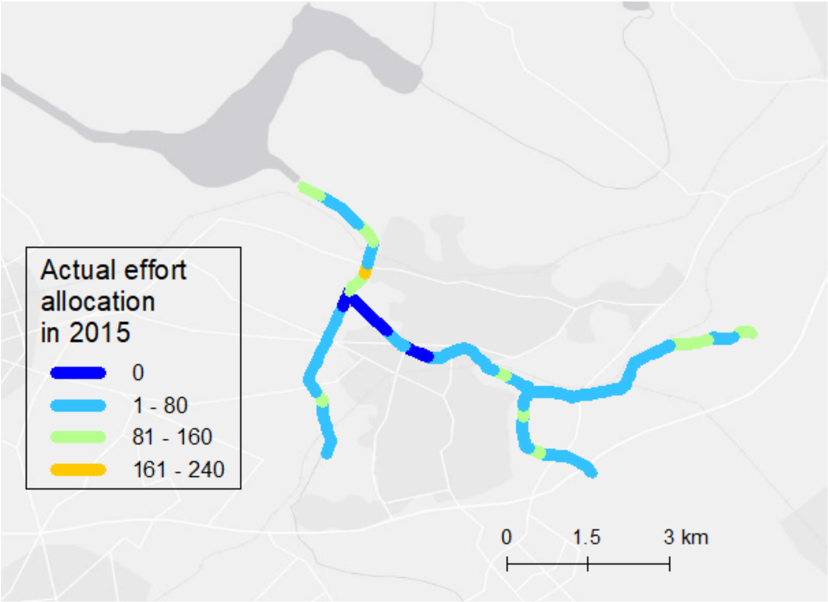

Fig. S1 (a9)

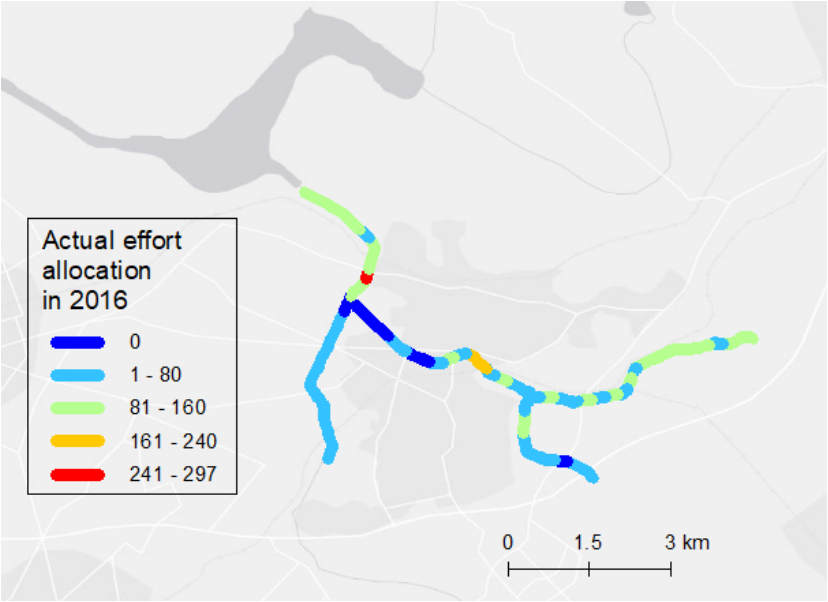

Fig. S1 (b1)

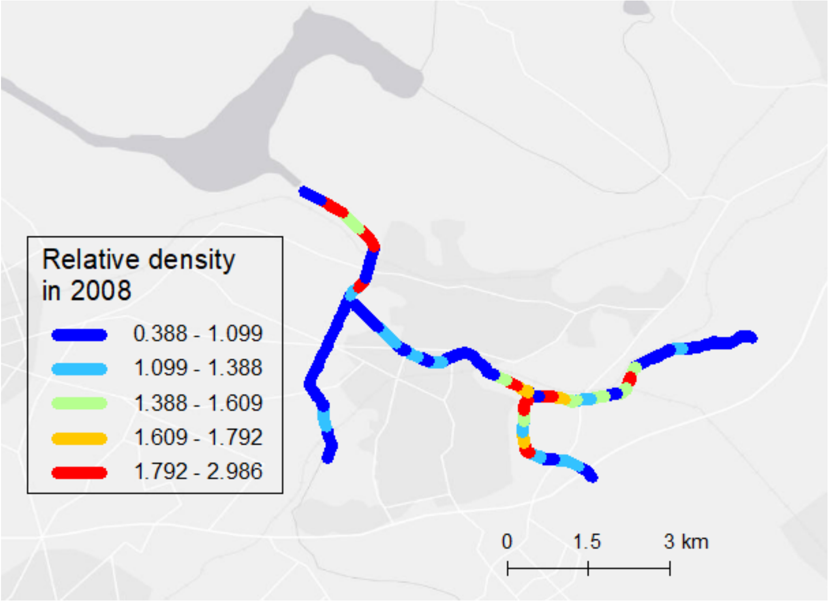

Fig. S1 (b2)

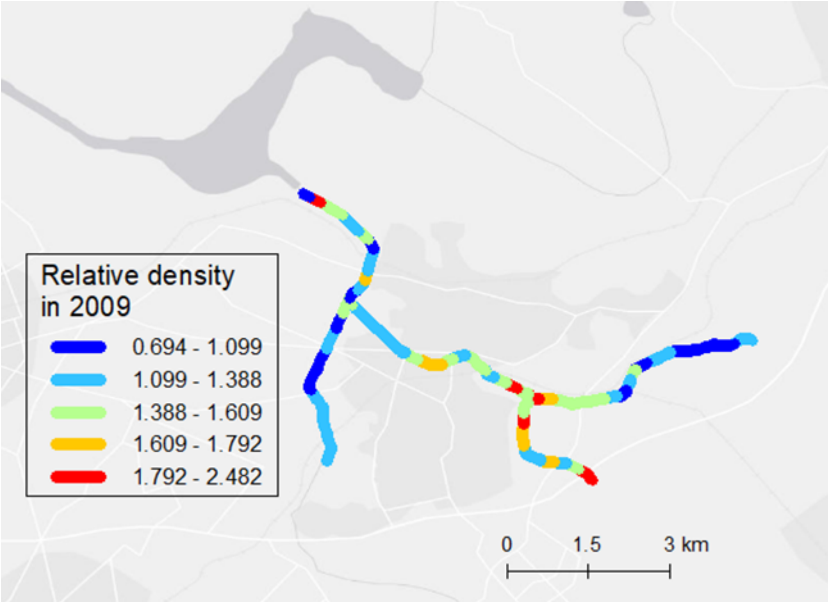

Fig. S1 (b3)

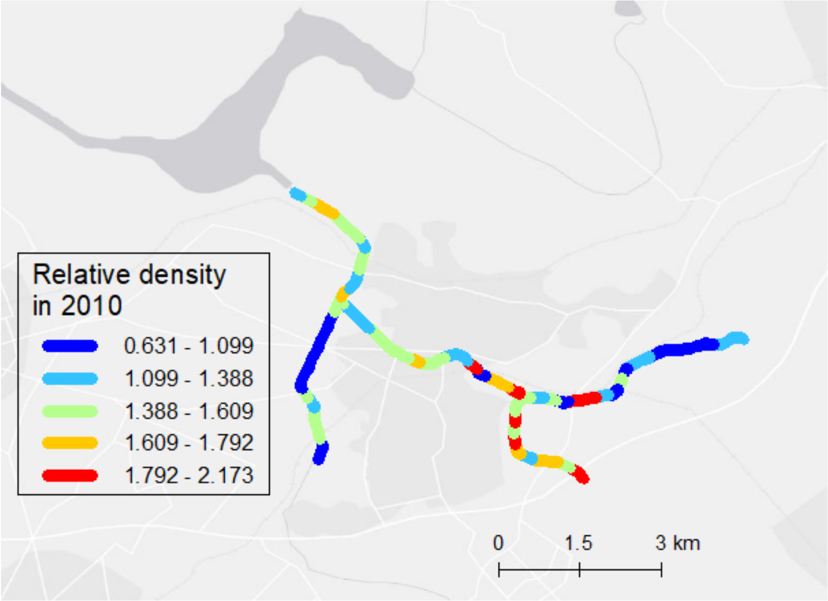

Fig. S1 (b4)

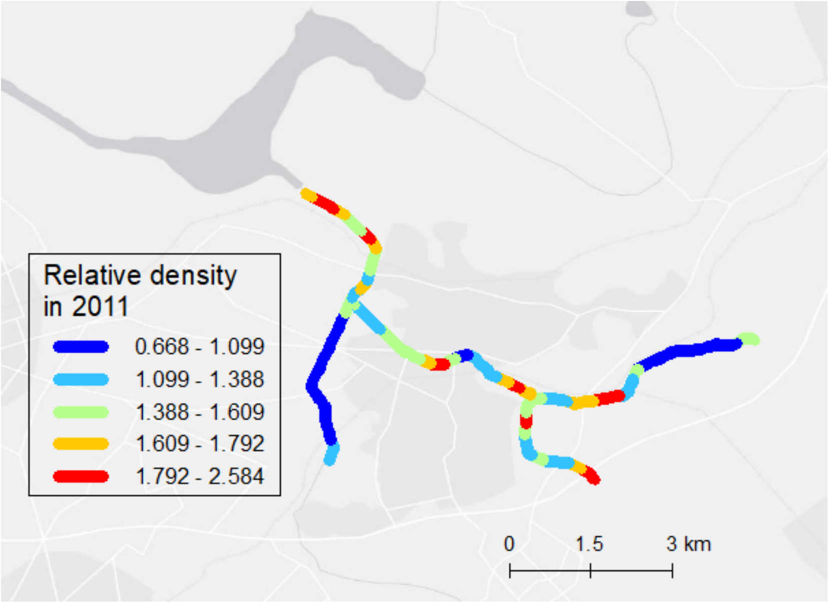

Fig. S1 (b5)

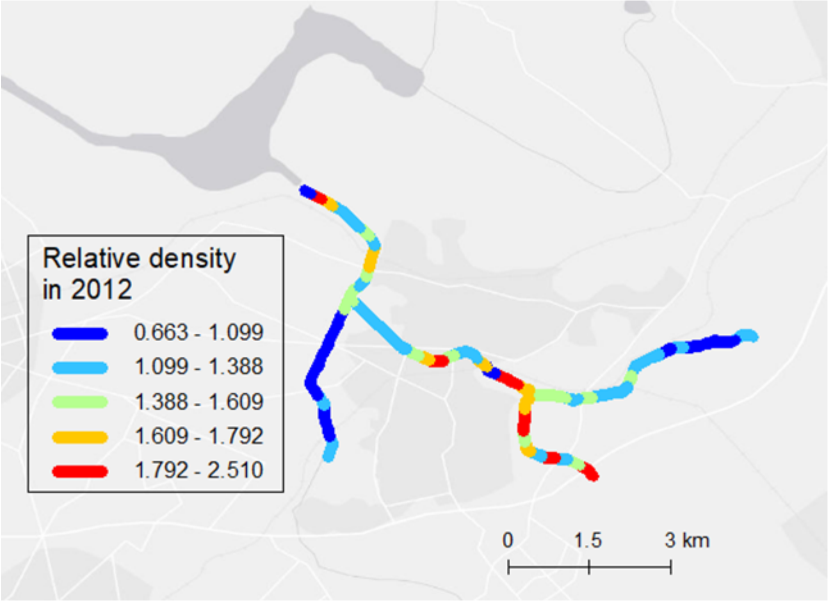

Fig. S1 (b6)

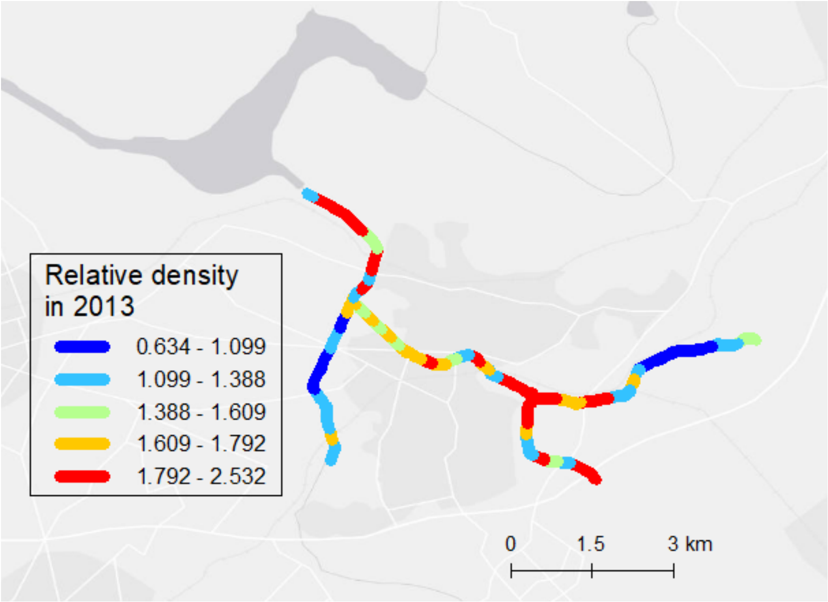

Fig. S1 (b7)

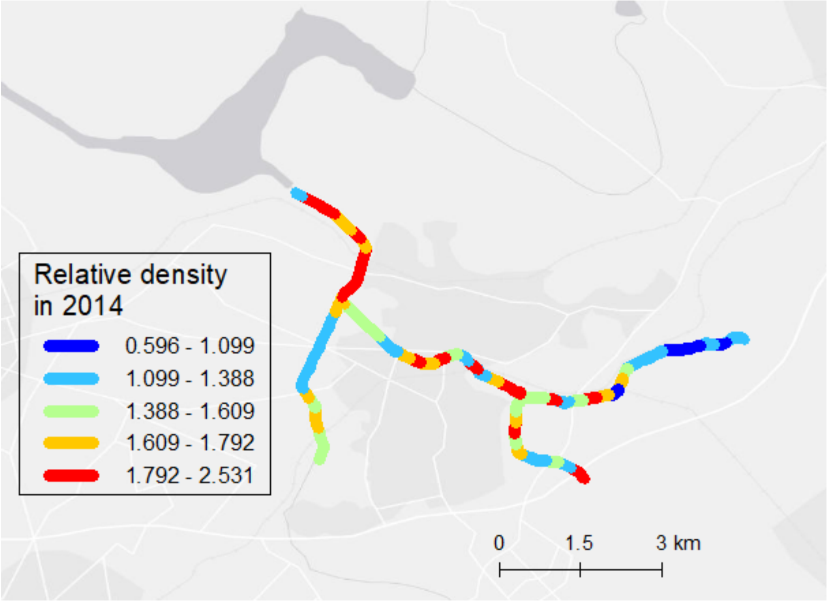

Fig. S1 (b8)

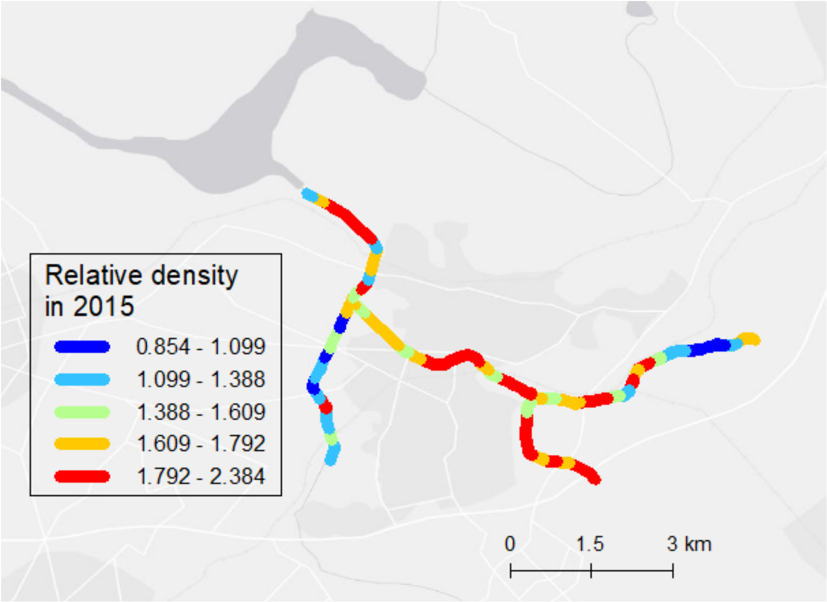

Fig. S1 (b9)

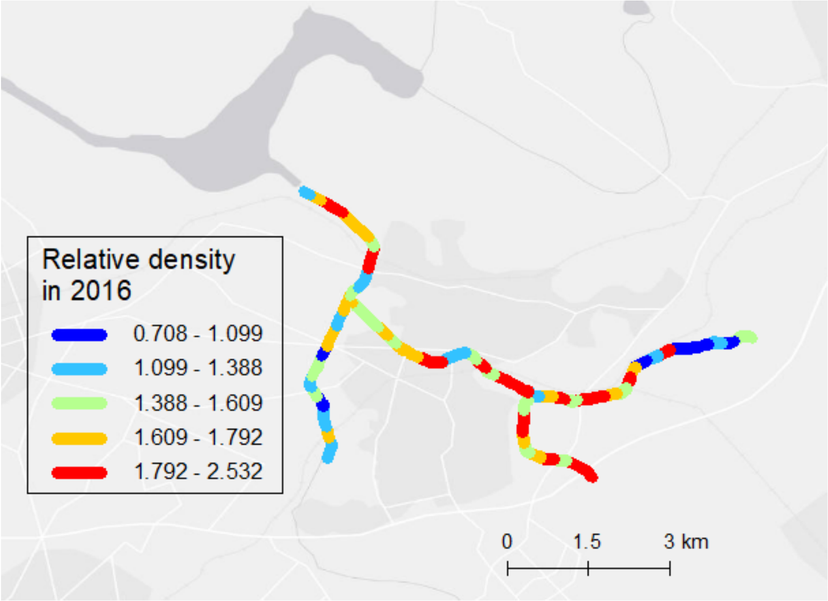

Figure S1. Spatial allocation in actual capture effort (100 trapping days) in (a1-9) 2008 to 2016 and the median relative density of snapping turtles in (b1-9) 2008 to 2016. [Background map source: Esri, HERE, Garmin, OpenStreetMap contributors, and the GIS user community].
