# Supplementary material for: Spatial optimization of invasive species control informed by management practices
Source: Ecol Appl. 2021 Jan 21;31(3):e02261. doi: 10.1002/eap.2261 (PMC8047888; doi:10.1002/eap.2261)
Supplement: Supplementary file 2 — Appendix S2 [file EAP-31-e02261-s004.pdf]

Supporting Information. Nishimoto, M., T. Miyashita, H. Yokomizo, H. Matsuda, T. Imazu, H. Takahashi, M. Hasegawa, and K. Fukasawa. 2020. Spatial optimization of invasive species control informed by management practices. Ecological Applications.

Appendix S2. Posterior predictive checks using Bayesian *p*-values

Table S1. Summary of posterior predictive checks for the total captured number in each year (quantiles of posterior predictive distribution, observed value and Bayesian *p*-value). If Bayesian *p*-value is near 0 or 1, there is large discrepancy between observation and the model prediction.

Year	2.5% CL	10% CL	50% CL	90% CL	97.5% CL	Observed value	Bayesian *p*-value
2008	114	125	146	168	182	156	0.714
2009	159	171	195	221	235	186	0.311
2010	145	157	179	203	217	174	0.377
2011	160	171	194	219	234	190	0.399
2012	137	148	170	194	207	164	0.348
2013	245	261	290	322	339	293	0.533
2014	277	293	325	359	377	331	0.570
2015	223	236	265	295	311	272	0.612
2016	244	259	288	320	335	292	0.559

CL: credible limit
