# Supplementary material for: Spatial optimization of invasive species control informed by management practices
Source: Ecol Appl. 2021 Jan 21;31(3):e02261. doi: 10.1002/eap.2261 (PMC8047888; doi:10.1002/eap.2261)
Supplement: Supplementary file 3 — Appendix S3 [file EAP-31-e02261-s001.pdf]

Supporting Information. Nishimoto, M., T. Miyashita, H. Yokomizo, H. Matsuda, T. Imazu, H. Takahashi, M. Hasegawa, and K. Fukasawa. 2020. Spatial optimization of invasive species control informed by management practices. Ecological Applications.

Appendix S3. Results of a simulation to investigating approaches for reaching equilibrium density

Fig. S1 (a1)

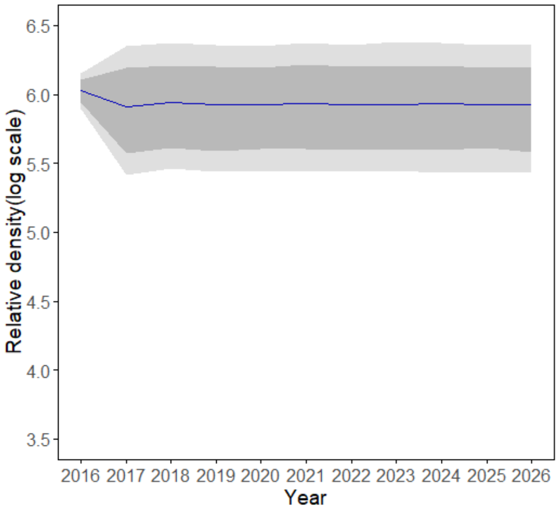

Fig. S1 (a2)

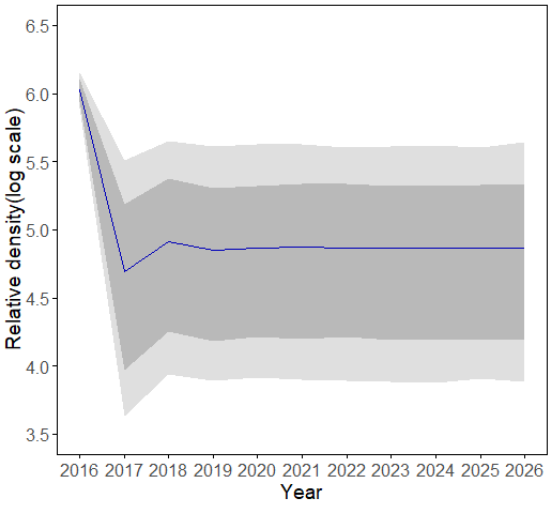

Fig. S1 (b1)

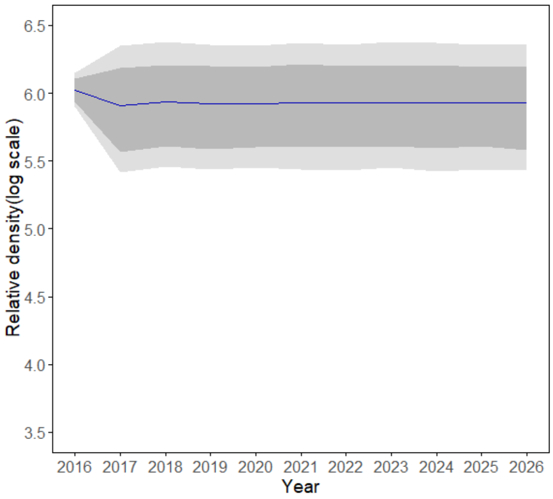

Fig. S1 (b2)

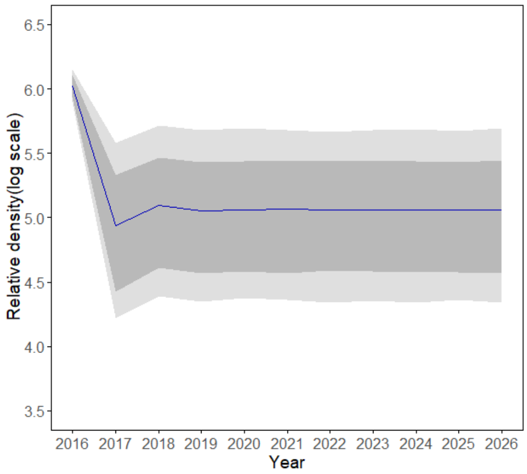

Fig. S1 (c1)

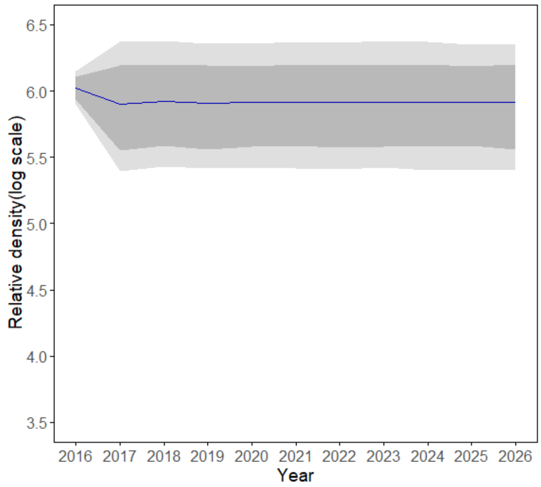

Fig. S1 (c2)

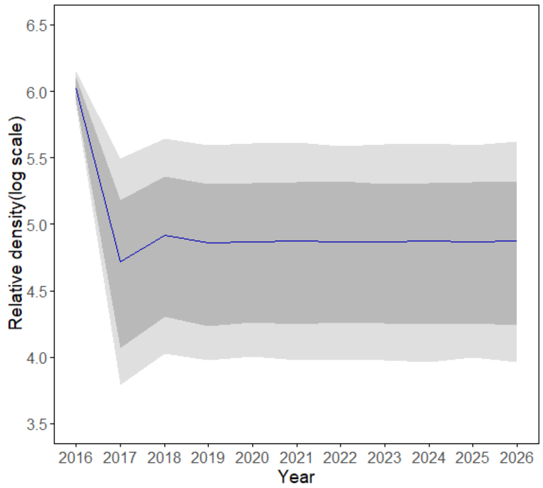

Figure S1. Results of a simulation investigating time to reach equilibrium density in each scenario. (a1-2) shows changes over time in relative density under two different management scenarios (1 and 8 times multiplications of the uniform allocation effort). (b1-2) shows changes over time in relative density under two different management scenarios (1 and 8 times multiplications of the current allocation effort in 2016). (c1-2) shows changes over time in relative density under two optimal scenarios by the simulated annealing method, given different total allocation effort (1 and 8 times of the

total effort amount). The base total amount of effort for (a)–(c) is the same as actual effort in 2016. The blue line indicates median relative density. The light gray band and a dark gray band indicate 95% CI (credible intervals) and 80% CI, respectively.
