# Supplementary material for: Spatial optimization of invasive species control informed by management practices
Source: Ecol Appl. 2021 Jan 21;31(3):e02261. doi: 10.1002/eap.2261 (PMC8047888; doi:10.1002/eap.2261)
Supplement: Supplementary file 4 — Appendix S4 [file EAP-31-e02261-s002.pdf]

Supporting Information. Nishimoto, M., T. Miyashita, H. Yokomizo, H. Matsuda, T. Imazu, H. Takahashi, M. Hasegawa, and K. Fukasawa. 2020. Spatial optimization of invasive species control informed by management practices. Ecological Applications.

Appendix S4. A comparison of greedy strategy and simulated annealing method in spatial effort allocation

We applied greedy algorithm (GrA) for searching solution that minimize the objective function and compared it with the results of simulated annealing (SA). In greedy strategy, the smallest unit of effort (1 trap-day) was allocated to the spatial unit with the highest equilibrium density and then the density was updated iteratively until the total effort amount was exhausted.

Table S1. Values of objective function (Obj) and mean relative densities (RD) under various total efforts in SA and GrA. "Multiplier of effort" indicates the multiplier of the total amount of effort in 2016.

Multiplier of effort	Obj in SA	Obj in GrA	RD in SA	RD in GrA	RD in GrA/ RD in SA
1	329.656	329.692	0.04395	0.04396	1.000
2	279.192	279.186	0.03723	0.03722	1.000
3	238.592	238.349	0.03181	0.03178	0.999
4	204.666	204.289	0.02729	0.02724	0.998
5	176.197	175.589	0.02349	0.02341	0.997
6	152.113	151.321	0.02028	0.02018	0.995
7	131.662	130.755	0.01755	0.01743	0.993
8	114.254	113.285	0.01523	0.01510	0.992

Obj in SA: value of objective function in simulated annealing allocation

Obj in GrA: value of objective function in greedy allocation

RD in SA: mean relative density in simulated annealing allocation

RD in GrA: mean relative density in greedy allocation

RD in GrA/ RD in SA: ratio of mean relative density in greedy allocation and simulated annealing allocation

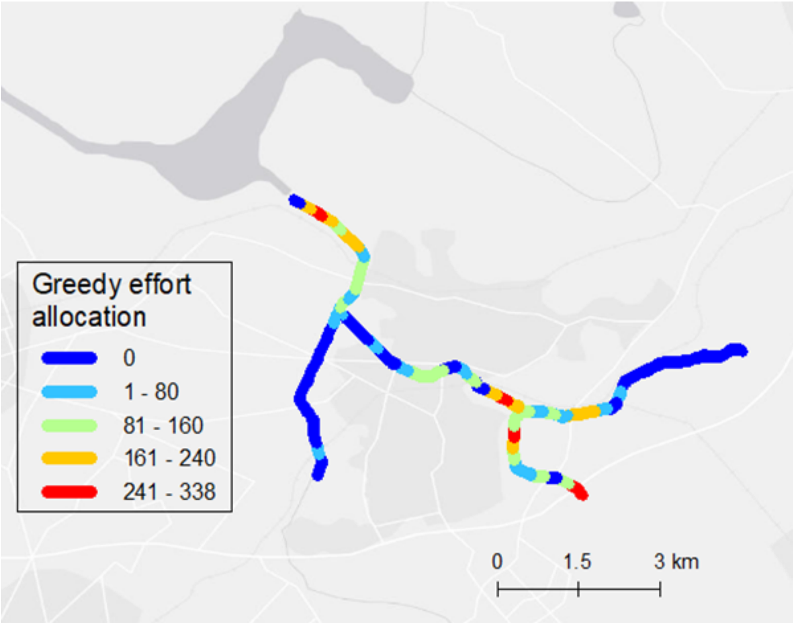

Figure S1. Maps showing the effort allocation (trap-days) calculated by greedy strategy. Total amount of effort is the same as actual effort in 2016. [Background map source: Esri, HERE, Garmin, OpenStreetMap contributors, and the GIS user community].
